# Lactose Breath Test: Possible Strategies to Optimize Test Performance, Accuracy, and Clinical Impact

**DOI:** 10.3390/nu16203516

**Published:** 2024-10-17

**Authors:** Giulia Scalese, Alessandra Cesarini, Lucia Pallotta, Emanuela Ribichini, Luca Spina, Maddalena Diofebi, Anna Citarella, Simona Cammarota, Carola Severi

**Affiliations:** 1 Gastroenterology Unit, Department of Translational and Precision Medicine, Sapienza University of Rome, 00185 Rome, Italy; giulia.scalese@uniroma1.it (G.S.); alessandra.cesarini@uniroma1.it (A.C.); lucia.pallotta@uniroma1.it (L.P.); emanuela.ribichini@uniroma1.it (E.R.);; 2 LinkHealth Health Economics, Outcomes & Epidemiology S.R.L., 80143 Naples, Italy; anna.citarella@linkhealth.it (A.C.); simona.cammarota@linkhealth.it (S.C.)

**Keywords:** lactose, malabsorption, intolerance, breath test

## Abstract

Lactose malabsorption (LM) refers to the incomplete absorption of lactose in the small intestine, resulting in the arrival of ingested lactose in the colon, which can give rise to symptoms defined as lactose intolerance (LI). The lactose breath test (LBT), thanks to its low cost, availability, and noninvasiveness, is the most used diagnostic method. However, the LBT is a tedious tool, requiring prolonged involvement of patients, qualified staff, and infrastructure, of which the most time-consuming factor is the frequency and number of breath samples needed. **Objectives**: To simplify the current LBT methodology, compliant with the current guidelines’ statements, by reducing the test duration or the number of breath samples, without compromising the test’s accuracy. **Methods**: The results of the standard LBT were compared with two simplified tests: a “shortened” test, lasting three hours, with samples taken every 30 min; and a “five-sample” test, lasting four hours, with samples taken every hour. Patients were stratified into three grades of malabsorption (mild, moderate, severe) based on the amount of gas exhaled. A clinical severity score was introduced to assess the clinical relevance of LI using a specific questionnaire. **Results**: Among the 543 patients enrolled (F 71.5%, mean age 43.7 ± 17.6 yrs), 60.4% (328/543) tested positive for LM. A total of 70.5% (383/543) presented LI, with 32.1% of those being true intolerants (LI without LM). The shortened test demonstrated an accuracy of 93.9%, with a sensitivity of 89.9% and a false negative rate of 10.1% (33/328). The five-sample test showed higher accuracy and sensitivity than the shortened test (96.5% and 94.2%, respectively; *p* = 0.03) with a false negative rate of 5.8% (19/328). Of the 19 false negatives in the five-sample test, 95% (18/19) were categorized as mild malabsorbents. No statistical correlation was found between the clinical severity score and LBT results. **Conclusions**: The five-sample test, involving hourly breath measurements, is a reliable option for simplifying the LBT without significantly reducing the procedure’s sensitivity.

## 1. Introduction

Breath tests are noninvasive diagnostic tools based on the measurement of hydrogen (H_2_) and methane (CH_4_), exclusively deriving from anaerobic fermentation of gut microbiota after the ingestion of test carbohydrates [1]. The lactose breath test (LBT) is probably the most commonly employed in clinical practice to diagnose lactose malabsorption (LM), which refers to the inability to digest lactose and/or absorb the products of its digestion in the small intestine [2]. Lactose is a common disaccharide found in dairy products, broken down by lactase-phlorizin hydrolase (LPH) into two monosaccharides, glucose and galactose, which are rapidly absorbed in the small intestine [3]. LM is caused by a primary or secondary lactase deficiency, characterized by a total or partial loss of synthesis and/or activity of LPH, normally expressed on the brush border of intestinal villi and responsible for the hydrolysis of the sugar [4,5]. In the case of LM, due to decreased lactose digestion and consequent absorption of the products of its hydrolysis, the undigested sugar reaches the colon, where the commensal flora metabolizes it into short-chain fatty acids (SCFAs), the osmotic effect of which may cause diarrhea [6,7]. At the same time, the presence of SCFAs in the lumen increases the colonic transit time and the contact time between the malabsorbed carbohydrate and the gut microbiota, with a consequent increase in the gas production of H_2_, CH_4_, and carbon dioxide (CO_2_) [8]. Gas causes a broad range of gastrointestinal (GI) symptoms, including abdominal pain, distension, bloating, and flatulence [9], collectively known as lactose intolerance (LI) [2]. During this process, the gases diffuse easily through the colonic mucosa into the bloodstream, reaching the lungs, where they are exhaled and can be detected for diagnostic purposes. In some patients, the predominant colonization by methanogenic archaea (e.g., *Methanobrevibacter smithii*) converts H_2_ into CH_4_, resulting in lower H_2_ and higher CH_4_ excretion. Low or non-emission of H_2_ can complicate the interpretation of LBT. LM cases may be misclassified as negative when only H_2_ is tested, resulting in an underestimation of the prevalence of LM [10,11].

Several tests are currently available to assess LM and LI, each examining different stages of the digestive and absorption process. Lactase deficiency/non-persistence can be diagnosed by directly measuring enzyme activity in mucosal biopsies or indirectly through genetic testing that evaluates the expression of the lactase gene [12,13]. The first method is rarely used due to its invasiveness and high cost, while the genetic test, limited to a few known modified polymorphisms typical of Caucasian patients, excludes many ethnic groups from the analysis and rules out the possibility of diagnosing secondary LM [5]. LM is detected using the LBT, and finally, LI is a clinical diagnosis based on the onset of symptoms during and/or after the test. The LBT remains the most widely used diagnostic technique due to its simplicity, noninvasive nature, and cost-effectiveness. However, the LBT has some drawbacks in clinical application due to its tedious procedure, which requires breath gas measurement every 30 min and can last between three and five hours.

Starting from their introduction in clinical practice, numerous efforts have been made to simplify the test and increase its accessibility for large-scale use, both trying to reduce the number of samples or the length of the entire test [14,15,16,17,18]. Unfortunately, the nature and dose of substrates, the diagnostic criteria, and the methodologies adopted in these previous works are extremely heterogeneous, making the results difficult to compare. These discrepancies have meant that previous suggested test modifications have never been applied in clinical practice. The recent publication of the European guidelines [19] provided exact statements about clinical indications, performance, and interpretation of the tests, giving the possibility to verify the reliability of previous results. Moreover, the guidelines highlighted the essential role of symptom assessment during the test, which represents the only available tool predictive of dietary efficacy [2]. LM and LI, in fact, refer to different aspects of the pathogenetic process, not necessarily connected, with different clinical relevance and therapeutic consequences. For this reason, the diagnosis of carbohydrate intolerance must be performed through a validated test-specific questionnaire [20]. Finally, the European guidelines recommend the measurement of CH_4_ in addition to H_2_ to increase LBT sensitivity in low or non-emitters of H_2_. 

The primary aim of this study was to simplify the current LBT methodology by either reducing the test duration or the number of breath samples, without compromising the procedure’s sensitivity. Secondly, starting from the new methodological aspects included in the European guideline and looking to improve the clinical utility of LBT, secondary objectives were to evaluate the clinical relevance of LI and assess the prevalence of CH_4_-producing microbiota.

## 2. Materials and Methods

### 2.1. Study Design 

From January 2022 to October 2023, 549 consecutive adult patients referred to the outpatient breath test service of the Gastroenterology Unit of the University Hospital Policlinico Umberto I, Sapienza University of Rome, for the H_2_/CH_4_ LBT for suspected LM were retrospectively enrolled in the study. The study was approved by the formal Ethical Committee, CET—Comitato Etico Territoriale Lazio Area 1 (Code: 7643), and conducted according to the ethical guidelines of the 1975 Declaration of Helsinki (6th revision, 2008). All patients provided informed consent.

### 2.2. Standard Test: Procedure and Diagnostic Criteria

The LBT methodology followed the current European guidelines [19]. Before performing LBT, all subjects received detailed instructions to prevent procedural errors. All patients were asked to abstain from antibiotics and laxatives for one month before the test and to delay testing for at least two weeks after colonic cleansing for endoscopic procedures. Probiotic intake was discontinued two weeks before the test and medications including antidiarrheals, opioids, prokinetics, and spasmolytics were discontinued for at least 48 h. The test was conducted after patients adhered to a 24 h low-FODMAP diet (fermentable oligosaccharides, disaccharides, monosaccharides, and polyols), which involved consuming low amounts of fruits, vegetables, grains, and bran cereals, followed by an 8 h fasting period and oral hygiene. Smoking and physical exercise were prohibited before and during the test, as hyperventilation can alter results. 

Nine breath samples were collected into plastic bags (Alfasigma^®^, Bologna, Italy) designed for sampling end-expiratory air, both before (time 0) and after administering a water solution containing 25 g of lactose. Samples were collected at 30 min intervals, up to 240 min. The concentrations of H_2_ and CH_4_ were measured in parts per million (ppm) using the Lactotest 202–Xtend (Medical Electronic Construction R&D srl, Brussels, Belgium), a diagnostic instrument that integrates three types of analyzers for H_2_, CH_4_, and CO_2_. Demographic and anthropometric data such as age, weight, and height were collected on the morning of the test. Clinical data on symptoms following the carbohydrate ingestion were recorded during the entire duration of the test, using the validated symptomatic questionnaire [20] to define the intolerance. Additionally, patients completed a self-assessed anamnestic symptom questionnaire designed by our research unit according to the Rome IV criteria [21] to assess the habitual GI symptomatology, namely, the clinical indications that led to the request for the test. Patients recorded the presence and frequency of bloating, diarrhea, and abdominal pain experienced in the preceding month and assigned a clinical severity score from 0 to 3 for each symptom: never, monthly, weekly, or daily. These scores were used to calculate the global clinical severity score.

The test was considered ‘positive’ if the gas peak exceeded baseline values by 20 ppm for H_2_ and/or by 10 ppm for CH_4_. Patients who tested positive under the standard test conditions were categorized as lactose malabsorptive; those exhibiting concurrent symptoms were classified as both malabsorptive and intolerant. Patients with negative test results but who experienced symptoms during the LBT were categorized as lactose intolerant.

### 2.3. Simplified Lactose Breath Test 

**Figure 1:**
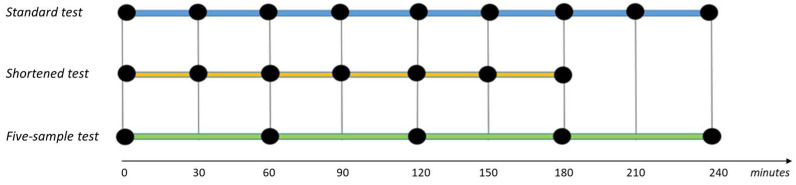
Test procedures of the three methodologies of lactose breath test. Each procedure was performed by administering 25 g of lactose. The standard test consists of nine breath samples, collected every 30 min up to 240 min (0, 30, 60, 90, 120, 150, 180, 210, 240). The shortened test consists of seven measurements, collected every 30 min up to 180 min (0, 30, 60, 90, 120, 150, 180), while the five-sample test consists of five measurements, collected every 60 min up to 240 min (0, 60, 120, 180, 240).

To simplify the current LBT methodology, results from the standard LBT were compared with those from two simplified tests: the “shortened” and the “five-sample” LBT. The shortened test involved reducing the test duration to three hours, with breath samples collected every 30 min up to 180 min (0, 30, 60, 90, 120, 150, 180 min). The five-sample test reduced the sampling rate to once every 60 min, with measurements taken up to 240 min (0, 60, 120, 180, 240 min) (Figure 1). In these simplified tests, subjects whose gas peaks exceeded baseline values by 20 ppm for H_2_ and/or 10 ppm for CH_4_ were considered “positive”.

To place the false negative outcomes resulting from the simplified LBTs, the delta-ppm (Δ-ppm) and the area under the curve (AUC) were calculated to classify patients into three grades of malabsorption (mild, moderate, and severe), determined using the pre-specified cut-off defined as the lower 30% and the upper 30% of the range of each of the two gases’ distributions. The Δ-ppm was calculated as the difference between the maximum ppm value obtained during the test and the baseline value. The AUC was calculated from baseline to 240 min and expressed in arbitrary units of ppm/h. 

### 2.4. Statistical Analysis

Statistical analysis was conducted using the R statistical platform. The baseline characteristics of the subjects were described as mean ± standard deviation for numeric variables, and as absolute frequencies and percentages for categorical variables. Between-group comparisons were performed using the Student’s *t*-test for continuous variables and the chi-square test for categorical variables. The correlation between Δ-ppm and the AUC was assessed using Spearman’s rank correlation coefficient. The sensitivity, false negative rate (1 − sensitivity), and accuracy of the shortened and five-sample tests were calculated, using the standard LBT as the gold standard. The corresponding 95% confidence intervals (CIs) were estimated using the normal approximation for proportions. Due to the inherent characteristics of the two alternative tests, specificity was always equal to 100%. The difference in sensitivities between the shortened and the five-sample LBT was evaluated using the McNemar test for paired proportions among subjects with lactose malabsorption.

## 3. Results

**Figure 2:**
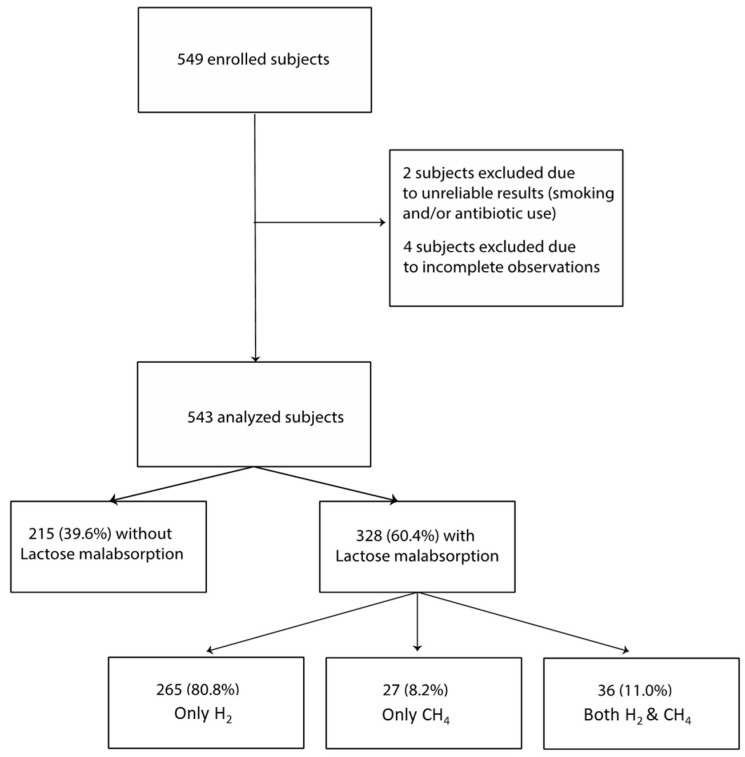
Flowchart of the study: from 549 subjects initially enrolled in the study, 543 patients were finally included in the analysis and lactose malabsorption was detected in 60.4% of patients. Abbreviations: H_2_: hydrogen, CH_4_: methane.

A total of 549 subjects were initially enrolled in the study. Six patients were excluded from the analysis: two due to unreliable results (one patient smoked during the test, and another had recently used antibiotics), and four due to incomplete observations. Thus, 543 patients were included in the final analysis. The mean age of the participants was 43.7 ± 17.6 years, with a female-to-male ratio of approximately 2:1 (388 F vs. 154 M). LM was detected in 60.4% (328/543) of patients: 80.8% with H_2_ excretion and 8.2% with CH_4_ (*p* < 0.0001). Only 11% of the positive tests showed excretion of both gases (Figure 2). Around 10% of malabsorption would have been lost without the concomitant measurement of H_2_ and CH_4_. Patients with LM were significantly older (44.7 ± 17.0 years) than those without (40.5 ± 17.6 years) (*p* = 0.006), with no differences in gender distribution.

### 3.1. Simplified LBT Validation

(a) Shortened test (reduction of test duration): Using the shortened test, the positivity rate was 54.3% (295/543); sensitivity was 89.9% (95% CI: 86.2% to 93.0%), and accuracy was 93.9% (95% CI: 91.6% to 95.8%). A total of 10.1% (33/328; 95% CI: 7.0% to 13.1%) of patients had a negative test, representing the false negative group.
(b) Five-sample test (reduction of the number of breath samplings): Using the five-sample test the positivity rate was 57% (309/543); sensitivity was 94.2% (95% CI: 91.1% to 96.1%), and accuracy was 96.5% (95% CI: 94.6% to 97.9%). The false negative rate was 5.8% (19/328).

### 3.2. Validation of the Five-Sample Test Through the Grades of Malabsorption

**Figure 3:**
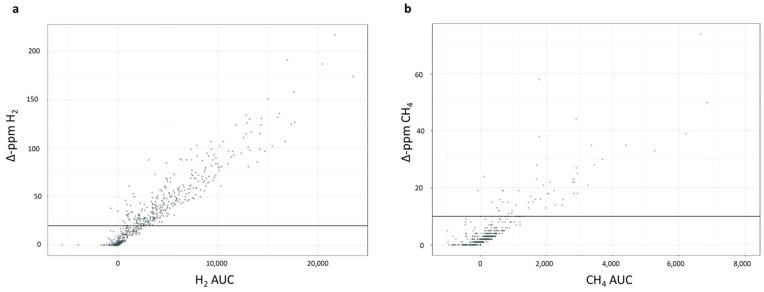
Correlation between Δ-ppm and AUC (**a**) for H_2_ and (**b**) for CH_4_. The Δ-ppm was calculated as the difference between the maximum value of ppm obtained during the test and the baseline value. The AUC was calculated from baseline to 240 min and expressed in arbitrary units of ppm/h. A statistically positive correlation was found between the Δ-ppm value and the AUC, both for H_2_ and CH_4_.

Given the higher sensitivity (*p* = 0.030) of the five-sample test compared to the shortened one, we analyzed the results obtained with this methodology to better characterize the false negative group. The relation between Δ-ppm and the AUC was calculated to identify the parameter for the categorization of subjects according to the severity of malabsorption. A statistically positive correlation was found between the Δ-ppm value and the AUC, both for H_2_ (r = 0.94) and CH_4_ (r = 0.78) (Figure 3a,b).

Since the Δ-ppm value is easily calculable in clinical practice, this parameter was used to stratify patients into three categories of malabsorption: mild, moderate, and severe (Table 1). Considering subjects with a diagnosis of LM according to H_2_ (*n* = 301), the majority (67.1%) had mild malabsorption, 30.9% had moderate malabsorption, and only 2% fell in the severe category. When the diagnosis was obtained using CH_4_, a similar pattern was observed, with the majority of subjects (58.7%) in the mild category, 36.5% in the moderate, and 4.8% in the severe one (Table 1).

**Figure 4:**
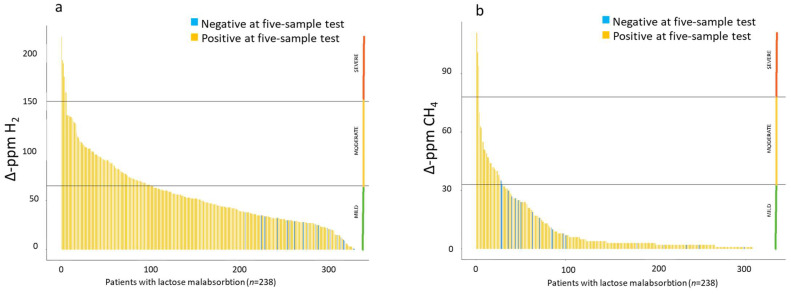
Waterfall plot of the Δ-ppm H_2_ (**a**) and CH_4_ (**b**) values in subjects with lactose malabsorption in the five-sample test. Horizontal lines represent the cut-off values of the three levels of malabsorption. Each vertical line represents a patient, the yellow lines indicate concordant results between the standard test and the five-sample test, while the blue lines indicate the false negative results obtained in the five-sample test. All the false negative results, except one positive for CH_4_, fall into the mild grade of malabsorption category.

Of the 19 false negative patients in the five-sample test, 13/19 (68.4%) were H_2_ producers, and 6/19 (31.6%) were CH_4_ producers. Almost all the false negative patients (95%) had mild malabsorption, except one (5%) who presented moderate malabsorption according to CH_4_, with Δ-ppm values slightly higher than the 30% cut-off (Figure 4a,b). Considering globally the positivity either of H_2_ or CH_4_, 73.7% (14/19) of false negative patients had symptoms during the test. 

### 3.3. Lactose Intolerance

The onset of symptoms, essential for the diagnosis of LI and its therapeutic implications, was assessed during the test. A total of 70.5% (383/543) of patients had at least one symptom, 67.9% (260/383) with LM and 32.1% (123/383) with a negative LBT, the latter constituting the group of only intolerants. LI was significantly more frequent in patients with LM than without it, 79.3% (260/328) and 57.2% (123/215), respectively (*p* < 0.0001). Finally, 20.7% (68/328) of LM patients were completely asymptomatic during testing, despite a positive result for malabsorption (Table 2). Therefore, approximately 21% of patients with malabsorption were not intolerant, and approximately 32% of intolerants were not malabsorptive.

### 3.4. Clinical Severity Score

**Figure 5:**
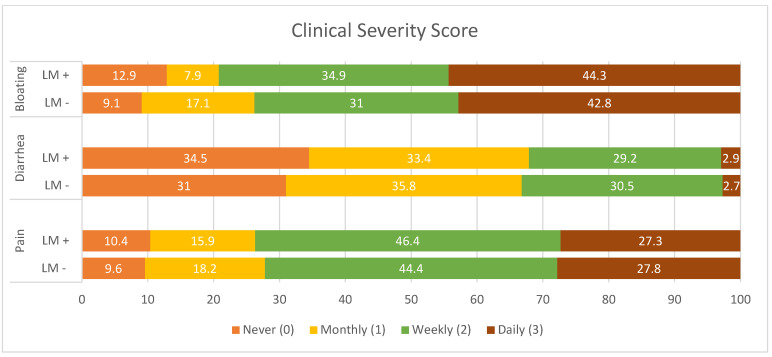
Clinical severity score. Clinical presentation was evaluated with a self-assessed questionnaire focused on symptoms experienced in the previous month. A score from 0 to 3 was assigned for each item: never: 0; monthly: 1; weekly: 2; daily: 3. Most patients reported a monthly occurrence of all the symptoms evaluated (pain, diarrhea, and bloating). Abbreviations. LM+: presence of lactose malabsorption, LM−: absence of lactose malabsorption.

The score was calculated through the self-assessed and shortened anamnestic symptom questionnaire to register GI symptoms reported by patients in the previous month, assigning a score from 0 to 3 based on the frequency of presentation (never, monthly, weekly, or daily) of abdominal pain, diarrhea, and bloating. Of the 543 patients enrolled in the study, 465 (85.6%) completed the self-assessed symptomatic questionnaire. All symptoms were mostly complained of at a weekly rate and abdominal pain was the most frequently reported (45.6%). Bloating was daily reported in 43.7% of patients (Figure 5). The clinical severity score showed no significant difference between patients with and without malabsorption (1.68 ± 0.65 vs. 1.67 ± 0.68, *p* = 0.92). No statistical correlation was found between the index and the Δ-ppm value of the gas exhaled (*p* = 0.61).

## 4. Discussion

The LBT is widely regarded as the preferred method for assessing LM and LI due to its noninvasive, cost-effective nature, simple methodology, and ability to detect both primary and secondary LM [2,19]. However, the main criticism of the LBT is its tedious methodology, primarily due to the lengthy procedure and frequent sampling requirements. These factors negatively impact patient compliance and increase the economic burden on healthcare facilities, which limits its widespread use in clinical practice. These criticisms were recognized almost immediately after its introduction into medical practice in the 1970s, so much so that over the years numerous attempts have been made to simplify the procedure by reducing testing time, the number of collected samples, and even modifying the quantity of substrate used [14,15,16,17,18,22,23,24,25]. However, the heterogeneity and discrepancies in the substrates used, the diagnostic criteria, and the methodologies adopted, did not permit their real application in clinical practice. A turning point in the management of breath testing was the recent publication of the European guidelines [19], which contributed to better-standardized clinical indications, test performance, and interpretation of results. 

Regarding test duration, the current recommendation is an interval of three to five hours [19], which can be adapted based on individual center experience. In 1986, Abramowitz and colleagues, in a mainly pediatric population, suggested the possibility of abbreviating the test to two hours, but using a lactose dose according to body weight, with a maximum of 50 g/kg, and adopting different diagnostic criteria to those proposed by the recent guidelines [14]. Several other studies attempting to shorten test duration using various substrate amounts have shown that reducing the test duration led to an underestimation of positive cases [22], as sensitivity dropped significantly to 74–54% with shorter test durations of 4, 3, or 2 h [22,23,24,25]. The loss of sensitivity was later suggested to be solved with the use of a high dose of lactose [15]. Positive evidence of the high accuracy of a short test, using the correct dose of lactose suggested by the European guidelines [19], came from a more recent study by Di Camillo and colleagues, involving over a thousand patients [16]. This study suggested that a three-sample test lasting up to three hours (0, 120, 180 min) achieved a sensitivity of 91.2%, with a further increase to 96.1% when the test was extended up to three and a half hours (0, 120, 210 min). The present study confirmed these last results, indeed the five-sample test with hourly breath measurements reaches a sensitivity of 94.2%. Shortening the test duration to 180 min leads to a significant drop in sensitivity to unacceptable levels, making it unsuitable as a replacement for the current standard methodology.

The problem of the lactose dose used during the test is crucial. The European guidelines recommend a lactose dose of 25 g because it provides enough substrate to produce sufficient H_2_ from bacterial fermentation, detectable in the breath, and triggers symptoms in most patients with clinically relevant carbohydrate intolerance [2,19]. Smaller lactose amounts lack sensitivity for LM, while larger quantities overestimate the prevalence of LI and possibly LM [18]. As previously commented, older studies obtained a good sensitivity for the short test only with higher lactose doses [15,17]. Even if the detection rates of LM and LI increase as the lactose dose increases [18], it should be kept in mind that 40–50 g of lactose corresponds to 1 L of fresh milk and is not representative of normal dietary intake. Furthermore, such a dose could induce symptoms even in healthy individuals without LM who can tolerate normal dietary lactose [2,26,27,28], thus likely promoting unnecessary and potentially harmful lactose-free diets. Given that the LBT is the only test to simultaneously assess LM and LI, the current guidelines recommend a lactose dose of 25 g to avoid an over-diagnosis of these conditions. Therefore, the dose of lactose recommended in the current guidelines does not allow for a safe reduction in test duration. 

The five-sample LBT, tested in the present study, proved to be an accurate strategy to optimize the procedure by increasing the interval between samples and reducing the total number of breath samples. Indeed, most false negative patients excluded from the five-sample test had mild lactose malabsorption, with only 16% showing a slightly higher grade of malabsorption. This suggests that the clinical presentation of these patients may not be primarily due to LM.

Indeed, speaking about the secondary aim, which is the assessment of the clinical relevance of LI, this study, consistent with a previous study [17], indicates that clinical symptoms do not correlate with LBT outcomes, resulting in equally distributed between patients with or without LM. The clinical severity score did not correlate with the degree of LM, and no significant differences were found between patients with and without LM, suggesting that heightened visceral sensitivity or other factors unrelated to LM may play a role in symptom development following carbohydrate ingestion. Many symptomatic patients suffer from disorders of gut–brain interaction (DGBI), particularly irritable bowel syndrome (IBS) [29], whose main driver is GI hypersensitivity [2]. In our study, true lactose intolerance (i.e., without LM) was observed in around 32% of patients, while almost 21% of patients with LM did not exhibit any gastrointestinal symptoms after lactose ingestion. Therefore, clinical presentation alone cannot reliably predict LM [19], and the LBT is crucial for diagnosing patients with LI who can effectively benefit from a lactose-free diet to alleviate gastrointestinal symptoms [30]. In this context, LBT results are clinically helpful in ruling out LI in unspecific GI disorders, whose management is rather complex.

Regarding the aim of evaluating the impact of CH_4_ emission, as reported in the previous literature, H_2_ was the principal gas exhaled. Adding the CH_4_ detection effectively increased the test sensitivity. In fact, in our cohort, despite the majority (91.8%) of malabsorptive patients being positive for H_2_, around 10% exhibited isolated CH_4_ positivity, which would have been undetected without measuring CH_4_. This aspect must be taken into account as in Western countries around 40% of the adult population are estimated to be CH_4_ emitters [31], and approximately 20% of the Central European population can even be classified as high-methane producers [32]. The simultaneous detection of multiple gases has been suggested as a factor contributing to the increased cost and complexity of the test [33], but actually, is not expected to increase the test’s cost and complexity because the equipment currently used in clinical practice reports both gases simultaneously, and some devices even offer dedicated software that automatically generates the final test report. Finally, consistent with the previous literature, our cohort showed an LM prevalence of approximately 60%, typical of Mediterranean countries [34,35], with LM diagnosis increasing with age, confirming that LPH expression decreases over time. 

The strengths of this study include its large sample size, which allowed access to analytical records and clinical data of 543 patients, as well as strict adherence to the methodology and diagnostic criteria outlined in the 2022 European guidelines. Further, the correct application of the guidelines allows the diagnosis of LI that could be helpful in dietary strategies for patients affected by DGBI. However, the exact cost-saving evaluation, in terms of both indirect and direct costs, required specific prospective studies focused on these health bioeconomic aspects, and represents a limitation of the study. 

## 5. Conclusions

In conclusion, when using the recommended dose of 25 g of lactose, only the five-sample test proved effective in optimizing the LBT without reducing test sensitivity compared to the shortened test. This substrate dose does not permit shortening the test to three hours without compromising sensitivity and accuracy. Extending the interval of breath sample collection with hourly measurements allows for a more efficient allocation of resources by reducing the involvement of qualified staff and maximizing the BT procedure, effectively doubling the number of patients who can be tested in a day.

## Figures and Tables

**Table 1:** The cut-off values for defining malabsorption severity and relative patient distribution.

		Mild Malabsorption	Moderate Malabsorption	Severe Malabsorption
**Δ-ppm H_2_**	**Cut-off value, ppm**	≤65	66–150	≥151
	**Patients, *n* (%)** **[total 301]**	202 (67.1%)	93 (30.9%)	6 (2%)
**Δ-ppm CH_4_**	**Cut-off value, ppm**	≤33	34–77	≥78
	**Patients, *n* (%)** **[total 63]**	37 (58.7%)	23 (36.5%)	3 (4.8%)

**Table 2:** Lactose intolerance in relation to the presence/absence of proven lactose malabsorption.

	Presence of Lactose Malabsorption (n = 328)	Absence of Lactose Malabsorption (n = 215)
Presence of GI symptoms during the test (n = 383)	260	123
Absence of GI symptoms during the test (n = 160)	68	92

## Data Availability

The data that support the findings of this study are available from the corresponding author, Carola Severi, upon reasonable request.
